# Feasibility and safety of the synchroseal articulating bipolar energy-based device for robotic gastrectomy in patients with gastric cancer: a prospective single-arm clinical trial with historical controls

**DOI:** 10.1007/s13304-025-02261-7

**Published:** 2025-08-04

**Authors:** Ji-Hyeon Park, JeeSun Kim, Danbi Lee, Seong-Ho Kong, Do Joong Park, Hyuk-Joon Lee, Han-Kwang Yang

**Affiliations:** 1 Department of Surgery, Seoul National University Hospital, Seoul, Korea; 2 Department of Surgery, Gachon University College of Medicine, Gachon University Gil Medical Center, Incheon, Korea; 3 Department of Surgery, Seoul National University College of Medicine, Seoul, Korea; 4 Cancer Research Institute, Seoul National University, Seoul, Korea; 5 VITCAL Co.,Ltd, Seoul, Korea

**Keywords:** Gastric cancer, Robotic gastrectomy, Articulating instrument, Energy-based devices, Bipolar, Ultrasonic

## Abstract

This study evaluated the feasibility and safety of Synchroseal (SS), a new articulating bipolar energy-based device, in da Vinci robotic gastrectomy for gastric cancer. A prospective study of 25 patients using SS was compared with retrospective data from 218 patients treated with conventional ultrasonic shears (US). Propensity score matching (PSM) ensured comparability. Metrics analyzed included C-reactive protein (CRP) levels, operative time, lymph nodes (LNs) retrieved, intraoperative blood loss, laboratory tests, hospital stay duration, and complication rates. PSM yielded a balanced comparison between the two groups (standardized differences < 0.1). SS (*n* = 25) significantly reduced CRP levels on postoperative days 2, 4, and 6 compared to US (*n* = 123) [7.67 ± 4.73 vs. 10.18 ± 5.66, (*p* = 0.040), 5.11 ± 3.33 vs. 6.65 ± 4.23, (*p* = 0.090), 2.74 ± 2.10 vs. 4.26 ± 3.78, (*p* = 0.001)]. Additionally, SS showed lower serum amylase levels and shorter operation times than US [67.60 ± 48.31 vs. 168.66 ± 316.92, (*p* = 0.027) and 234.52 ± 65.03 vs. 274.75 ± 54.90, (*p* = 0.002)]. Although SS retrieved fewer total LNs (31.80 ± 9.5 vs. 36.88 ± 14.96, *p* = 0.034), both groups achieved adequate LN dissection (> 30 LNs). No significant differences were observed in other parameters. SS led to lower postoperative CRP and serum amylase levels, shorter operation time, and adequate LN dissection, suggesting reduced postoperative inflammation and faster sealing function as potential benefits.

## Introduction

Minimally invasive surgery (laparoscopic/robotic) is becoming the standard of surgery for gastric cancer in Korea [1–3]. According to the 2019 National Gastric Cancer Surgery Survey, led by the Korean Society of Gastric Cancer, the number of minimally invasive gastric cancer surgeries in Korea has steadily increased since 2004 (6.6%), accounting for 80.3% of all gastric cancer surgeries performed in Korea in 2023 [4]. The proportion of robotic gastrectomy compared to laparoscopic gastrectomy has shown a steady increase over the past decade, rising from 2.1% in 2014 to 5.6% in 2019, and reaching 9.5% in 2023, according to the latest nationwide survey conducted by the Korean Gastric Cancer Association. Robotic surgery continues to gain popularity as it offers advantages such as tremor-free precision and articulated instrument movement with more degrees of freedom, resembling that of the human hand, which help overcome the technical limitations of conventional laparoscopic procedures [4–6].

Therefore, all surgical instruments used in robotic surgery are designed with articulation capabilities, except energy-based devices (EBDs), which play an important role in minimally invasive surgeries. This is because adding articulated capabilities to EBDs using an ultrasonic system is technically difficult and has not yet been achieved. Therefore, in the real world, outdated nonarticulated ultrasonic shears (US), which was used in laparoscopic surgery a long time ago, is still widely used in robotic surgery.

The articulation function was successfully added to EBDs using a bipolar system. In a previous study, we evaluated the feasibility of the 1 st generation articulating bipolar EBD, called the Vessel sealer, in da Vinci robot-assisted gastrectomy [7]. The results showed lower high-sensitivity C-reactive protein (hsCRP) levels and higher albumin levels with the Vessel sealer than with conventional US. Although the Vessel sealer provided a good configuration in the direction of dissection owing to its articulation function, it was somewhat challenging to perform sharp or delicate dissection. This is because the vessel sealer was designed with a thick jaw and blunt tip, as the bipolar system requires a separate cutting system after coagulation.

Synchroseal® (Intuitive Surgical, Inc., Sunnyvale, CA USA) is the most recently developed robotic bipolar EBD that addresses the limitations of conventional robotic EBDs by incorporating both a sharp tip and articulation functionality. In a previous study, it was possible to create a sharp tip because the Synchroseal (SS) was not equipped with a separate cutting blade, even though it supplied heat energy through the bipolar system [8]. This new articulating bipolar EBD with a sharp tip might be beneficial in performing a precise and appropriate lymph node (LN) dissection, which is one of the most important factors for ensuring oncological safety in gastric cancer surgeries [9, 10].

Therefore, in this study, we aimed to evaluate the feasibility and safety of the new articulating bipolar EBD, the SS, in da Vinci robotic gastrectomy with a focus on its impact on intraoperative inflammatory response and short-term surgical outcomes.

## Materials and methods

### Study design and ethical statement

This prospective single-arm clinical trial with external controls. The experimental group, enrolled at Seoul National University Hospital (SNUH) from September 2021 to December 2021, underwent robotic gastrectomy using SS, while the external control arm consisted of retrospective data from SNUH (November 2016 to December 2021). Propensity score matching (PSM) ensured unbiased estimation [11].

The study received approval from the Institutional Review Board of SNUH (IRB No. D-2108-182-1248). Informed consent was obtained from the experimental group, and consent requirements for the retrospective control group were waived due to the study's retrospective nature. The study adhered to Good Clinical Practice and the Declaration of Helsinki.

### Primary and secondary endpoints

The primary outcome was serum hsCRP, a surrogate marker for complications and inflammation, levels on postoperative day (POD) 2. Secondary outcome measures included intraoperative blood loss (IBL), operation time, number of examined LNs, length of hospital stay (LoS), serum laboratory tests on POD 2, 4, 6, and 14, including hsCRP, and postoperative complications.

### Sample size calculation for experimental group

The sample size was calculated using G*Power 3.1.9.7 (Heinrich-Heine-Universität Düsseldorf, Düsseldorf, Germany) based on our earlier robot-assisted distal gastrectomy study [7]. In the previous study, the mean and standard deviation of hsCRP at POD 2 for ultrasonic and bipolar EBDs were 11.5 ± 6.6 and 8.0 ± 2.8, respectively [7]. Based on these results, the calculated effect size was 0.7. Using a two-tailed *t* test with *α* = 0.05 and *ß* = 0.2, the minimum numbers of patients for detecting differences between the two groups were calculated to be 25 (power = 81%) for the experimental group.

### Participants

Robotic gastrectomy is planned for the patients with following conditions in routine clinical practice of SNUH: (1) adults aged 20 years or older; (2) pathologically proven gastric adenocarcinoma (3) clinically T1–4, N ±, and M0 on preoperative gastroscopy and abdominal computed tomography (CT) according to the most recent update of the American Joint Committee on Cancer/Union for International Cancer Control (AJCC/UICC) TNM staging system; (4) expected R0 resection with curative intent by robotic gastrectomy; (5) when the potential of open conversion during the operation is minimal after review of medical, psychosocial, and previous surgical history.

Patients who were determined to be candidates for robotic gastrectomy in routine clinical practice and who voluntarily agreed to use SS as an EBD during the whole surgical procedure instead of conventional US by written informed consent and preoperative hsCRP < 1.0 mg/dl, were enrolled in the experimental group.

In the external control group, data were collected retrospectively from patients who underwent robotic gastrectomy using conventional US. Among the 218 patients who underwent robotic gastrectomy during the study period, those who did not have information on IBL (*n* = 10), preoperative hsCRP (*n* = 48), or EBD used during surgery (*n* = 3) were excluded. Patients who underwent robotic wedge resection or open conversion (*n* = 5) were also excluded. A total of 152 patients were included in the PSM (Fig. 2).

### Surgeons

SNUH is a high-volume center in Korea that performs 700–800 gastric cancer surgeries annually. The four participating gastrointestinal surgeons were experts with more than 10 years of experience in gastric cancer surgery. Each of them performs more than 100 gastric cancer-related gastrectomies (open/laparoscopic/robotic) per year, of which at least 2–4 cases per month are robotic gastrectomies. To reduce bias in the learning curve of the SS device, each surgeon performed two pilot cases using the SS before the clinical trial to familiarize themselves with its use. Robotic gastrectomies performed without enrollment in the experimental arm during the entire study period were performed using conventional US.

### Intervention and operative procedures

In the experimental arm, conventional robotic gastrectomy (total, distal, proximal, or pylorus-preserving gastrectomy) with D1 + or D2 LN dissection was performed using SS unless the surgeon decided to discontinue its use due to safety concerns. Except for the use of SS, all other surgical procedures, including anesthesia, patient positioning, port insertion, robot docking, and instrument placement, were performed in the usual manner of robotic gastrectomy performed at SNUH [7]. All reconstruction approaches (intracorporeal/extracorporeal) and methods (Billroth I, Billroth II, Roux-en-Y, double tract, and gastrogastrostomy) related to each gastrectomy procedure were allowed.

### Pre-, intra-, and post-operative measurements and data collection

In the experimental arm, pre-, intra-, and post-operative data were prospectively collected. Clinicopathological information, such as age, sex, preoperative body mass index, and American Society of Anesthesiology (ASA) score, was collected preoperatively. Information on the operation and reconstruction methods, IBL, blood transfusion volume, and operation time was collected during the operation. For the accurate measurement of IBL, SNUH routinely weighs fresh gauze before the operation and used gauze after the operation in robotic gastrectomy and calculates differences. The IBL was defined as the sum of the blood volume calculated from the used gauze and the blood volume in the suction bottle. Serum laboratory tests, including hsCRP, were performed preoperatively on POD 2, 4, 6, and 14 days after discharge. The amount of Jackson-Pratt (JP) drainage was measured from POD 1 to POD 7. Serum and drain amylase levels were measured on POD 2. The pathological results, including the number of retrieved LNs, were collected postoperatively. Adverse reactions and complications were observed and assessed intra- and post-operatively. Discharge was planned within POD 5–10 unless an extension of admission was needed owing to adverse reactions. Postoperative complications were counted until POD 30, including the first outpatient visit 2 weeks after discharge.

For the external control group, the same relevant data as those for the experimental group were collected retrospectively.

### PSM and statistical analysis

Continuous and categorical variables are summarized as the means ± standard deviations (SDs) and proportions/percentages, respectively.

PSM was performed using R software (version 4.0.0; R Foundation for Statistical Computing, Vienna, Austria) to align the clinicopathological information of patients in the single and external arms. PSM was used to estimate the average marginal effect of the'SS'on ‘CRP of POD 2’ on those who received it accounting for confounding by the included covariates: Age, sex, preoperative body mass index, medical history, operator, and operation name. The propensity score was calculated using a prohibit regression model, and patients in the two groups were matched using full matching with a caliper width of 0.25 SD. Absolute standard differences were used to evaluate the balance of the confounding variables between the two groups after PSM.

Demographics, intra-, and post-operative outcomes between two groups were analyzed using the χ-square test and Fisher’s exact tests for categorical variables and independent *t* test for continuous variables. The IBM SPSS Statistics ver. 26 (IBM Corp., Armonk, NY, USA) was used for the analysis. Statistical significance was set at *P* value < 0.05.

## Results

### Patients and baseline characteristics

**Fig. 1:**
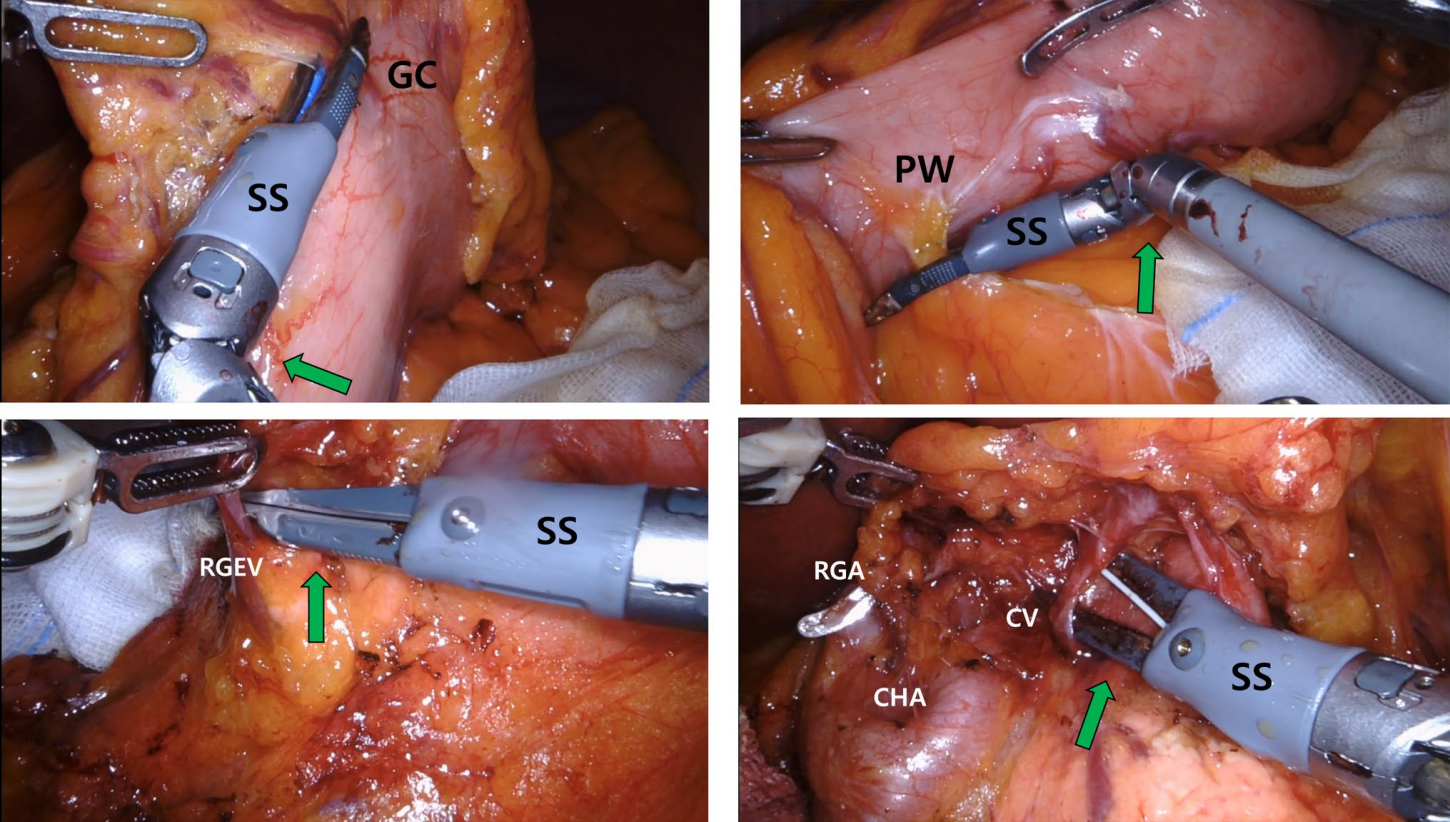
Robot gastrectomy with Synchroseal. **a** Articulated movement. **b** Lymph node dissection with refined, curved jaw. *SS* Synchroseal, *GC* greater curvature, *PW* posterior wall, *RGEV* right gastroepiploic vein, *RGA* right gastric artery, *CV* coronary vein, *CHA* common hepatic artery

**Fig. 2:**
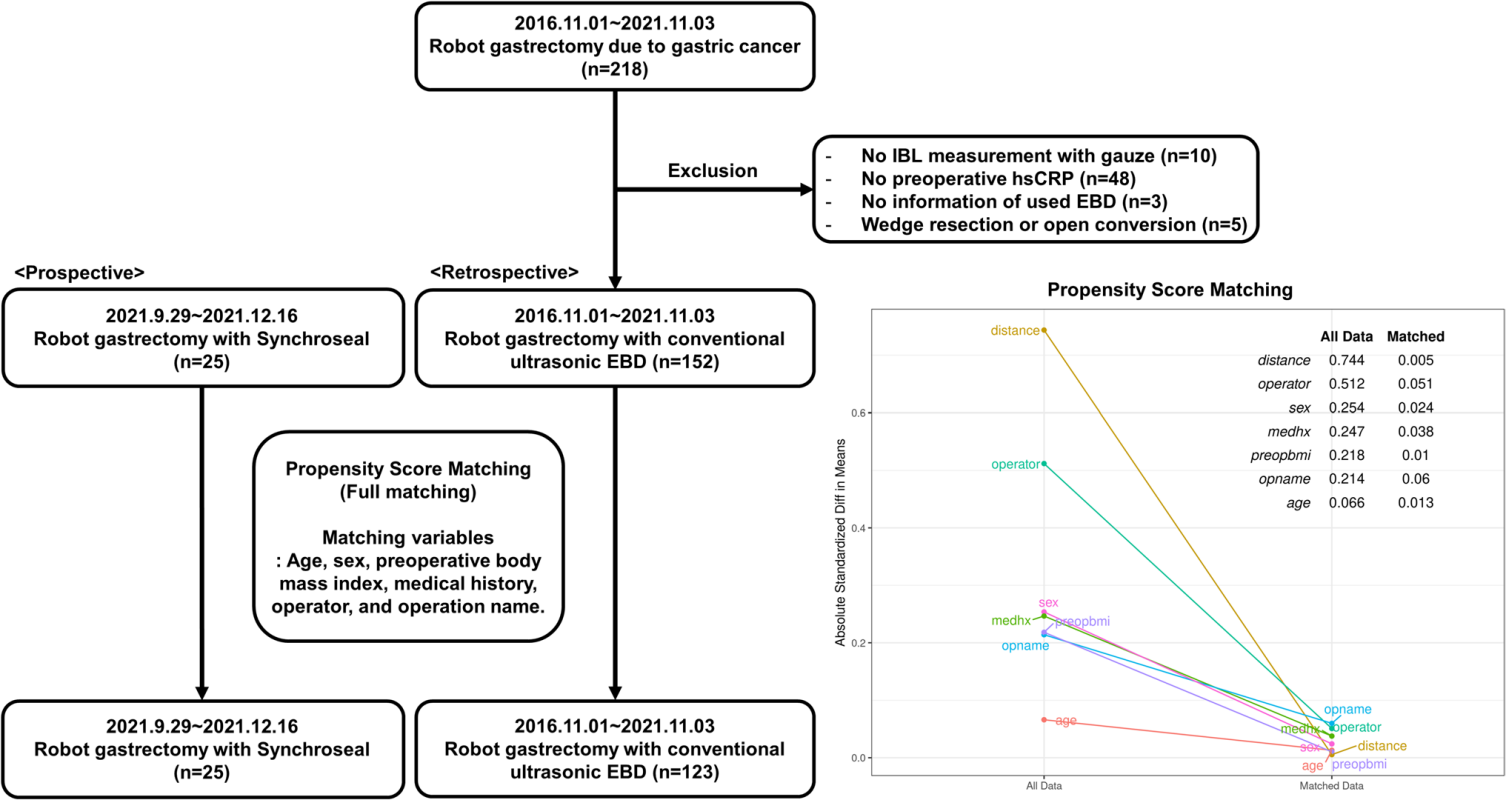
Study flow diagram **a** Patient enrollment **b** Propensity score matching condition. *IBL* Intraoperative blood loss, *hsCRP* high-sensitivity C-reactive protein, *EBD* energy-based device

As planned, 25 patients were prospectively enrolled in the SS group during the study period. Robotic gastrectomy with standard LN dissection was performed in all patients (Fig. 1). There were no cases in which SS was switched to conventional US in the middle of the surgery owing to safety issues. In total, 123 patients were selected from the external control group using conventional US after PSM. Full matching of propensity scores between the two groups yielded an adequate balance, as shown in Fig. 2. After matching, the absolute standardized mean differences for all covariates were less than 0.1. As shown in Table 1, the patients were well-matched and showed similar clinicopathological characteristics between the groups.

**Table 1:** Patient demographics and baseline characteristics

Variable	SS (*n* = 25)	US (*n* = 123)	Effect estimate (95% CI)	*p* value
Age at operation date, years	58.68 ± 14.72 (25)	58.80 ± 11.31 (123)	−0.12 (−5.29 to 5.06)	0.964^a^
BMI, kg/m^2^	23.40 ± 3.26 (25)	23.76 ± 2.89 (123)	−0.36 (−1.64 to 0.92)	0.575^a^
Sex			0.82 (0.35 to 1.94)	0.652^b*^
Male	13 (52.0%)	70 (56.9%)		
Female	12 (48.0%)	53 (43.1%)		
Past medical history			1.38 (0.58 to 3.28)	0.464^b^
No	14 (56.0%)	59 (48.0%)		
Yes	11 (44.0%)	64 (52.0%)		
ASA score				
2	25 (100.0%)	120 (100.0%)		
Tumor location				0.493^b^
Upper third	3 (12.0%)	25 (20.3%)		
Middle third	10 (40.0%)	37 (30.1%)		
Lower third	12 (48.0%)	61 (49.6%)		
ESD			0.49 (0.09 to 2.69)	0.340^c^
No	23 (92.0%)	117 (95.9%)		
Yes	2 (8.0%)	5 (4.1%)		
Clinical T stage				0.682^c^
T1	22 (88.0%)	93 (75.6%)		
T2	2 (8.0%)	18 (14.6%)		
T3	1 (4.0%)	11 (8.9%)		
T4	0 (0.0%)	1 (0.8%)		
Clinical N stage			1.36 (0.29 to 6.44)	1.000^c^
N0	23 (92.0%)	110 (89.4%)		
N−/+	2 (8.0%)	13 (10.6%)		
Pathology stage (AJCC 8 th)				0.777^c^
I	21 (84.0%)	107 (87.0%)		
II	3 (12.0%)	12 (9.8%)		
III	1 (4.0%)	4 (3.3%)		
Type of resection				0.485^c^
Distal gastrectomy	10 (40.0%)	59 (48.0%)		
Total gastrectomy	1 (4.0%)	10 (8.1%)		
Pylorus-preserving gastrectomy	12 (48.0%)	38 (30.9%)		
Proximal gastrectomy	2 (8.0%)	16 (13.0%)		
Anastomosis method			0.72 (0.30 to 1.74)	0.467^b^
Extracorporeal	10 (40.0%)	59 (48.0%)		
Intracorporeal	15 (60.0%)	64 (52.0%)		
Type of reconstruction				0.559^c^
Billroth I	6 (24.0%)	24 (19.5%)		
Billroth II	4 (16.0%)	34 (27.6%)		
Roux-en-Y Gastrojejunosotmy	0 (0.0%)	1 (0.8%)		
Roux-en-Y Esophagojejunostomy	1 (4.0%)	10 (8.1%)		
Double tract	2 (8.0%)	16 (13.0%)		
Gastrogastrostomy	12 (48.0%)	38 (30.9%)		
LN dissection			1.78 (0.49 to 6.43)	0.570^c^
D1 +	22 (88.0%)	99 (80.5%)		
D2	3 (12.0%)	24 (19.5%)		
Combined resection				0.602^c^
None	25 (100.0%)	116 (94.3%)		
Yes (Gallbladder)	0 (0.0%)	7 (5.7%)		

Data are reported as the mean ± standard deviation (*n*) for continuous variables and *n* (%) for categorical variables

Effect estimates are presented as mean differences for continuous variables and as odds ratios for categorical variables, with corresponding 95% confidence intervals (CIs)

*SS* Synchroseal, *US* ultrasonic shears, *BMI* body mass index, *ASA* American society of anesthesiologists score, *ESD* endoscopic submucosal dissection, *AJCC* American Joint Committee on Cancer, *LN* lymph node

Statistical analysis: ^a^t-test, ^b^Chi-square test, ^C^Fisher’s Exact Test

*p < 0.05

## Outcomes

1. Primary endpoint

**Fig. 3:**
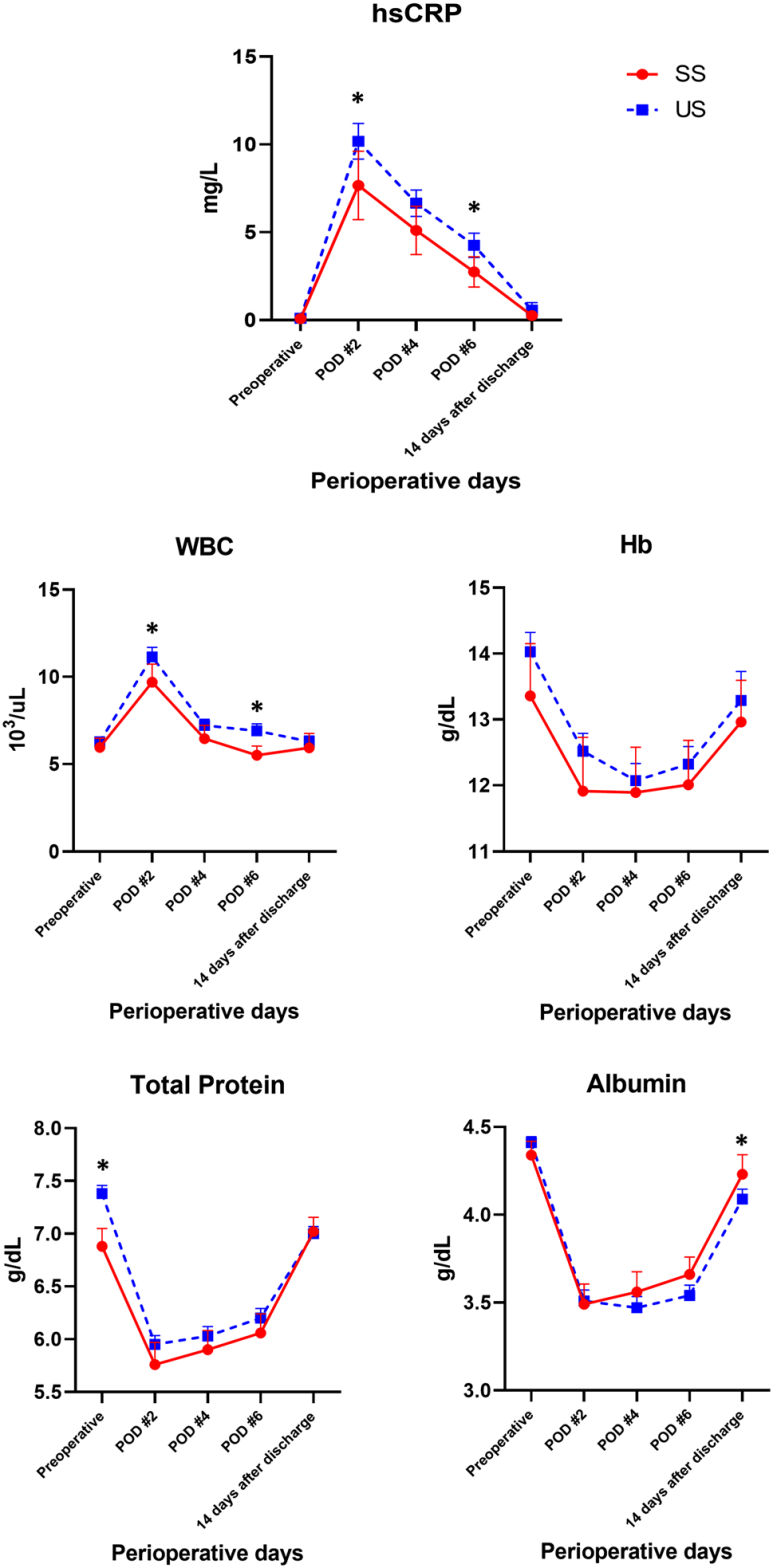
Change in serum laboratory result **a** hsCRP, **b** WBC, **c** Hemoglobin, **d** Total protein, and **e** Albumin. *SS* Synchroseal, *US* Ultrasonic shears, *POD* postoperative day, *hsCRP* high-sensitivity C-reactive protein, *WBC* white blood cell, *Hb* hemoglobin. Error bars indicate 95% confidence intervals. *p < 0.05

At POD 2, the SS group had significantly lower hsCRP levels and lower white blood cell (WBC) levels compared to the US group [7.67 ± 4.73 ml vs 10.18 ± 5.66 ml (*p* = 0.040) for hsCRP; 9.69 ± 2.52 ml vs 11.14 ± 3.13 ml (*p* = 0.030) for WBC] (Fig. 3a and 3b). The hsCRP levels peaked on POD 2 and continued to decrease to the baseline until 14 days after discharge. During this time, hsCRP levels on POD 4 and 6 were lower in the SS group compared to the US group [5.11 ± 3.33 ml vs 6.65 ± 4.23 ml (*p* = 0.090) on POD 4; and 2.74 ± 2.10 ml vs 4.26 ± 3.78 ml (*p* = 0.007) on POD 6]. This pattern was seen with WBC counts [6.45 ± 1.87 ml vs 7.22 ± 1.92 ml (*p* = 0.067) on POD 4; 5.50 ± 1.30 ml vs 6.90 ± 2.23 ml (*p* = 0.000) on POD 6]. There were no clinically significant differences between the groups in other laboratory tests, including hemoglobin, total protein, and albumin levels (Fig. 3c–e).

(2) Secondary endpoints

As shown in Table 2, the operation time in SS was significantly shorter than that in the US (234.52 ± 65.03 min vs. 274.75 ± 54.90 min, *p* = 0.002). Although the SS group had a higher IBL (42.23 ± 53.20 ml vs. 25.44 ± 22.07 ml, *p* = 0.133), it was not statistically significant. There were no cases of intraoperative blood transfusions in either group. There was no significant difference in the amount of JP drainage between the two groups on PODs 1–7. The serum and drain amylase level on POD 2 were significantly lower in SS compared to US [67.60 ± 48.31 vs. 168.66 ± 316.92 (*p* = 0.050) for serum amylase; 235.72 ± 246.21 vs. 401.08 ± 480.58 (*p* = 0.050) for drain amylase]. However, there was no statistically significant differences in Drain/Serum amylase on POD 2 (3.84 ± 4.23 vs. 3.67 ± 3.03, *p* = 0.853). Although the SS group had a shorter postoperative LoS (10.24 ± 3.19 days vs. 12.28 ± 7.44 days, *p* = 0.180), it was not statistically significant. Complication rate and severity appeared to be higher in the US group than in the SS group; however, this difference was not statistically significant. There was one case of postoperative ileus (Clavien-Dindo grade < IIIa) in the SS group. This was resolved with conservative management.

**Table 2:** Surgical outcomes and complications

Variables	SS (*n* = 25)	US (*n* = 123)	Effect estimate (95% CI)	*p* value
Operation time, minutes	234.52 ± 65.03 (25)	274.75 ± 54.90 (123)	−40.23 (−64.81 to −15.65)	0.002^a^
IBL, ml	42.23 ± 53.20 (25)	25.44 ± 22.07 (123)	16.79 (−5.47 to 39.05)	0.133^a^
Intraoperative transfusion				
No	25 (100%)	123 (100%)		
Yes	0	0		
JP drainage amount				
POD 1	117.88 ± 62.40 (25)	122.61 ± 76.32 (123)	−4.74 (−36.91 to 27.44)	0.772^a^
POD 2	116.82 ± 71.14 (25)	114.75 ± 89.63 (123)	2.07 (−35.59 to 39.73)	0.914^a^
POD 3	101.68 ± 60.40 (25)	116.37 ± 88.08 (123)	−14.69 (−51.18 to 21.80)	0.427^a^
POD 4	86.63 ± 65.54 (22)	104.88 ± 88.18 (119)	−18.25 (−57.32 to 20.82)	0.357^a^
POD 5	66.83 ± 64.35 (17)	91.51 ± 86.06 (88)	−24.68 (−68.33 to 18.96)	0.265^a^
POD 6	61.67 ± 37.47 (9)	84.52 ± 75.76 (48)	−22.85 (−74.89 to 29.18)	0.383^a^
POD 7	61.67 ± 46.46 (3)	90.46 ± 80.81 (28)	−28.80 (−126.85 to 69.26)	0.553^a^
JP drainage amylase on POD 2, U/L	235.72 ± 246.21 (25)	401.08 ± 480.58 (52)	−165.36 (−330.44 to −0.27)	0.050^a^
Serum amylase on POD 2, U/L	67.60 ± 48.31 (25)	168.66 ± 316.92 (53)	−101.06 (−190.36 to −11.76)	0.027^a^
Drain/Serum amylase on POD 2, U/L	3.84 ± 4.23 (25)	3.67 ± 3.03 (52)	0.18 (−1.50 to 1.85)	0.855^a^
Postoperative hospital stay, days	10.24 ± 3.19 (25)	12.28 ± 7.44 (123)	−2.05 (−5.05 to 0.96)	0.180^a^
Complications			2.84 (0.35 to 22.73)	0.466^b^
No	24 (96.0)	110 (89.4)		
Yes	1 (4.0)	13 (10.6)		
Types of complications				0.643^b^
Postoperative ileus	1 (4.0)	2 (1.6)		
Anastomosis site leakage	0 (0.0)	3 (2.4)		
Pancreatic fistula	0 (0.0)	1 (0.8)		
Postoperative fluid collection	0 (0.0)	5 (4.1)		
Anastomosis site stenosis	0 (0.0)	2 (1.6)		
C–D grade				0.286^b^
< IIIa	1 (1.8)	3 (3.3)		
≥ IIIa	0 (1.8)	10 (1.7)		

Data are reported as the mean ± standard deviation (*n*) for continuous variables and *n* (%) for categorical variables

Effect estimates are presented as mean differences for continuous variables and as odds ratios for categorical variables, with corresponding 95% confidence intervals (CIs)

*SS* Synchroseal, *US* ultrasonic shears, *IBL* intraoperative blood loss, *JP* Jackson-Pratt, *C*–*D* Clavien–Dindo

Statistical analysis: ^a^t-test, ^b^Fisher’s Exact Test

*p < 0.05

More number of LNs were retrieved in US compared to SS (31.80 ± 9.5 for SS, vs 36.88 ± 14.96 ml for US, *p* = 0.034) (Table 3). When it was compared by each LN station, there were fewer number of LNs were retrieved in SS compared to US in LN station #6 (3.13 ± 2.60 vs. 5.32 ± 3.44, *p* = 0.005), #7 (3.32 ± 2.93 vs. 5.15 ± 3.31, *p* = 0.011), and #8a (2.32 ± 1.41 vs. 3.12 ± 2.35, *p* = 0.027).

**Table 3:** Pathologic results

Variable	SS (*n* = 25)	US (*n* = 123)	Mean difference (95% CI)	*p* value
Total number of retrieved LNs	31.80 ± 9.5 (25)	36.88 ± 14.96 (123)	−5.08 (−9.75 to −0.40)	0.034^a^
**#1**	2.56 ± 2.35 (25)	3.86 ± 3.51 (119)	−1.30 (−2.75 to 0.16)	0.080^a^
**#2**	2.00 ± 1.73 (3)	2.54 ± 2.10 (28)	−0.54 (−3.12 to 2.04)	0.674^a^
**#3**	6.88 ± 6.11 (25)	5.47 ± 4.55 (122)	1.41 (−1.22 to 4.05)	0.282^a^
**#4 sa**	1.67 ± 2.08 (3)	0.96 ± 1.78 (26)	0.71 (−1.55 to 2.96)	0.526^a^
**#4 sb**	1.08 ± 1.75 (25)	1.56 ± 2.90 (119)	−0.48 (−1.68 to 0.71)	0.425^a^
**#4 d**	7.00 ± 3.60 (22)	6.32 ± 4.08 (111)	0.69 (−1.17 to 2.54)	0.466^a^
**#5**	1.00 ± 1.79 (11)	0.92 ± 1.15 (73)	0.08 (−0.72 to 0.89)	0.839^a^
**#6**	3.13 ± 2.60 (23)	5.32 ± 3.44 (107)	−2.19 (−3.69 to −0.68)	0.005^a^
**#7**	3.32 ± 2.93 (25)	5.15 ± 3.31 (122)	−1.83 (−3.24 to −0.42)	0.011^a^
**#8a**	2.32 ± 1.41 (25)	3.12 ± 2.35 (122)	−0.80 (−1.51 to −0.10)	0.027^a^
**#9**	3.20 ± 2.36 (25)	3.19 ± 2.63 (118)	0.01 (−1.12 to 1.13)	0.993^a^
**#10**	- (0)	0.50 ± 0.58 (4)		
**#11p**	2.15 ± 1.84 (20)	2.41 ± 2.31 (108)	−0.26 (−1.34 to 0.82)	0.638^a^
**#11 d**	1.00 (1)	1.08 ± 1.38 (13)	−0.08 (−3.20 to 3.05)	0.958^a^
**#12a**	0.33 ± 0.58 (3)	1.28 ± 1.54 (25)	−0.95 (−2.82 to 0.92)	0.308^a^

Data are reported as the mean ± standard deviation (*n*) for continuous variables and *n* (%) for categorical variables

*SS* Synchroseal, *US* ultrasonic shears, *LN* lymph node

Statistical analysis: ^a^t-test

*p < 0.05

## Discussion

As the number of robotic gastric cancer surgeries increases, there is a growing need for articulated EBD that enables delicate, comfortable, and safe robotic gastrectomy. Recently, Da Vinci introduced SS, an articulated bipolar EBD with a refined, curved jaw. However, the effectiveness of robotic gastrectomies using this EBD for treating gastric cancer has not yet been reported. This study was conducted in 25 patients undergoing different types of robotic gastrectomy to provide substantial evidence of the feasibility and safety of SS by comparing postoperative outcomes with those of patients using conventional US.

The novelty of SS is that it not only allows for full wrist movement but also provides a single-step seal-and-cut using radiofrequency energy [8]. This one-step capability eliminates the need for a separate cutting device, allowing the design of a thin curved jaw despite being a bipolar system. Our previous study showed that the Vessel sealer, the 1 st generation robotic bipolar EBD, has the advantage of low collateral thermal damage; however, because of its thick and blunt jaws, it is not often used in gastric cancer surgery, where delicate and safe LN dissection is important [7]. Therefore, we expect that this one-step sealing and cutting capability would be a major benefit for the entire surgery. However, early in our study, even though the operators were quite pleased with quick sealing, they complained that when they tried to cut through thin fat or tissue after sealing, the cut did not seem to go through properly. This dissatisfaction decreased as the number of surgeries using SS increased. In addition, as noted in the results, in all 25 patients who underwent gastrectomy with SS, there was no single case of SS being replaced with other EBDs owing to intraoperative safety concerns.

The most important function of an EBD is to securely seal the blood and lymphatic vessels with minimal collateral thermal damage [12, 13]. Our research team has many experiences in comparing and analyzing different types of EBDs in gastric cancer surgery [7, 14, 15], and we have obtained clinically meaningful results by examining blood serum hsCRP and cytokine levels to check the postoperative inflammatory response caused by collateral thermal damage, and serum and drain amylase levels to check for pancreatic injury.

Given the relatively low incidence of major complications following gastric cancer surgery, surrogate biochemical markers are often employed to detect subtle differences in surgical stress and tissue response. Among them, C-reactive protein (CRP) is widely recognized as a clinically useful marker that reflects the magnitude of postoperative inflammation, including tissue trauma induced by thermal energy during surgery. In particular, its perioperative trends have been explored in relation to postoperative morbidity.

Previous studies have applied CRP-based indicators—such as absolute values, time-point comparisons, and composite scoring systems—to predict early infectious or severe complications following gastrectomy. These approaches have demonstrated that CRP levels can aid in the early identification of patients at risk and may be especially relevant in the context of minimally invasive or energy device-assisted surgeries [16–20].

Based on our experience with a number of similar studies, the primary endpoint of this study was to compare postoperative hsCRP levels, a routine clinical marker of the systemic inflammatory response after elective surgery [21, 22]. In line with our earlier findings that bipolar EBDs result in less collateral thermal damage than ultrasonic or hybrid devices [7, 15], we also found that the SS group had significantly lower postoperative hsCRP levels and WBC counts than the US group on POD 2, 4, and 6. These results support the idea that SynchroSeal may help reduce postoperative systemic inflammation, likely due to its superior thermal sealing characteristics.

In gastric cancer surgery, the risk of pancreatic injury during suprapancreatic LN dissection (LN stations 7, 8, 9,1 1p, 11 d) is high, and this may cause serious postoperative complications, such as a pancreatic fistula; therefore, surgeons are careful to minimize pancreatic injury when dissecting these LN stations. Intraoperative pancreatic injury can be caused by an assistant compressing the pancreas to facilitate the operator's LN dissection, but it can also be caused by collateral thermal damage from the EBDs [23]. In our previous study, we expected that intraoperative pancreatic injury would be significantly lower in patients with bipolar EBDs because the outer part of the jaw that heats and cuts the vessel or tissue is insulated. However, the results showed that although postoperative serum amylase levels were the lowest with the bipolar EBD, this was not statistically significant. Drain amylase levels are even higher than those in other EBDs [15]. However, in this study, as expected, both serum and drain amylase levels were lower with SS than with US, with serum amylase being statistically significant. In the case of US, one case of pancreatic fistula was reported as a complication, although there was a bias due to the earlier time of surgery and a larger number of patients in the US group compared to SS. Although this study was limited by time-interval bias, both in this study and in our previous studies, hsCRP, WBC, and serum amylase levels were lower in bipolar EBDs than in other EBDs, suggesting that bipolar EBDs have less collateral thermal injury than other EBDs.

In previous studies comparing different laparoscopic EBDs in gastric surgery, bipolar EBD had shorter operative time [7, 15, 24, 25]. In this study, the operative time in the SS group was also significantly shorter than that in the US group, which may be a result of its faster sealing time due to its one-step sealing and cutting capability. As mentioned by Kim et al. omitting vessel clipping during small-vessel ligation may also affect the operative time [24]. However, as this was not a randomized controlled trial, the two EBDs were not used concurrently during the study period. Although this study also included some patients who underwent robotic gastrectomy using conventional US during the same study period, the possibility of bias cannot be excluded because most of the control group using US underwent gastrectomy much earlier period than the SS group by the same four surgeons. Therefore, the operative time advantage needs to be validated in future well-designed randomized controlled trials.

IBL and postoperative hemoglobin levels were checked to determine the hemostatic capability of SS, which was introduced to seal blood vessels and tissue bundles up to 5 mm in diameter that fit the jaws of the instrument. In previous studies, there was slightly less IBL with robotic and laparoscopic bipolar EBDs than with other EBDs [7, 15, 24]. Unexpectedly, the SS group had slightly more intraoperative bleeding in this study, but the difference was not statistically significant compared with the US group. This may be due to the learning curve, as each surgeon performed only two pilot cases using SS before starting the study. None of the groups received any intraoperative blood transfusions. During the postoperative hospital stay, both groups maintained postoperative hemoglobin levels above 10 g/dL. These results indicated that both EBDs were acceptable in terms of hemostatic function.

Adequate LN dissection is essential for radical gastrectomy. The appropriate total number of retrieved LNs in gastric cancer surgery is controversial, ranging from LNs > 15 to as many as LNs > 30 in the literature [26–28]. In the present study, the total number of LNs detected in both groups was > 30; however, the number of LNs detected using US was significantly higher than that detected using SS. We believe that despite being a bipolar system, the jaw of the SS is sharp, pointed, and articulated (Fig. 1), making it a much more delicate and easier LNs dissection. However, when we performed a subgroup analysis according to the LN station, the number of LNs retrieved by US was significantly higher at LNs 6, 7, and 8. Although this result was somewhat disappointing compared with our expectations, the overall number of retrieved LNs was greater than adequate, suggesting that LN dissection with SS is comparable to that with other EBDs.

This study has the limitation of being a single-center, single-arm prospective study with a small sample size. In Korea, although the proportion of robotic surgeries for gastric cancer is gradually increasing, broader clinical adoption remains limited due to constraints in insurance coverage. Consequently, conducting large-scale prospective studies using robot-specific EBDs such as SS remains challenging. To our knowledge, studies evaluating articulating EBDs like SS in robotic gastrectomy are extremely limited. To partially address this limitation, we performed a PSM comparison with an external control group treated with conventional US, aiming to reduce selection bias and improve comparability. Nevertheless, we fully acknowledge that these study design limitations remain, and more definitive conclusions regarding the clinical efficacy and long-term surgical outcomes associated with SS should be validated through future randomized controlled trials.

In conclusion, this study examined the sealing function, collateral thermal injury, and adequacy of LN dissection with the SS to determine whether it was a new EBD that retained the advantages of the conventional bipolar EBDs while addressing its disadvantages. SS showed no difference in IBL or postoperative hemoglobin levels and lower postoperative hsCRP, WBC, and serum amylase levels compared to US, indicating a comparable sealing function with less collateral thermal injury. It demonstrated a shorter operation time with good configuration in the direction of dissection owing to its articulation function and delicate dissection with a sharp tip. It also had a comparable complication rate, suggesting adequate safety. These findings may help guide surgeons in selecting an appropriate EBD for robotic gastrectomy.

## Data Availability

The data that support the findings of this study are available from the corresponding author, Han-Kwang Yang, upon reasonable request.
